# A new method for *in vivo* assessment of corneal transparency using spectral-domain OCT

**DOI:** 10.1371/journal.pone.0291613

**Published:** 2023-10-05

**Authors:** Maëlle Vilbert, Romain Bocheux, Cristina Georgeon, Vincent Borderie, Pascal Pernot, Kristina Irsch, Karsten Plamann

**Affiliations:** 1 Laboratory for Optics and Biosciences (LOB)— École Polytechnique, CNRS, INSERM, IPP, Palaiseau, France; 2 Vision Institute—CNRS, INSERM, Sorbonne University, Paris, France; 3 GRC 32, Transplantation et Thérapies Innovantes de la Cornée, Sorbonne Université, Centre Hospitalier National d’Ophtalmologie des Quinze-Vingts, Paris, France; 4 Physical Chemistry Institute (ICP)—CNRS, University of Paris-Saclay, Orsay, France; 5 LOA—ENSTA Paris, École polytechnique, CNRS, IPP, Palaiseau, France; National Institute of Laser Enhanced Sciences (NILES), Cairo University, EGYPT

## Abstract

Corneal transparency is essential to provide a clear view into and out of the eye, yet clinical means to assess such transparency are extremely limited and usually involve a subjective grading of visible opacities by means of slit-lamp biomicroscopy. Here, we describe an automated algorithm allowing extraction of quantitative corneal transparency parameters with standard clinical spectral-domain optical coherence tomography (SD-OCT). Our algorithm employs a novel pre-processing procedure to standardize SD-OCT image analysis and to numerically correct common instrumental artifacts before extracting mean intensity stromal-depth (*z*) profiles over a 6-mm-wide corneal area. The *z*-profiles are analyzed using our previously developed objective method that derives quantitative transparency parameters directly related to the physics of light propagation in tissues. Tissular heterogeneity is quantified by the Birge ratio *B*_*r*_ and the photon mean-free path (*l*_*s*_) is determined for homogeneous tissues (i.e., *B*_*r*_*~1*). SD-OCT images of 83 normal corneas (ages 22–50 years) from a standard SD-OCT device (RTVue-XR Avanti, Optovue Inc.) were processed to establish a normative dataset of transparency values. After confirming stromal homogeneity (*B*_*r*_ <10), we measured a median *l*_*s*_ of 570 μm (interdecile range: 270–2400 μm). By also considering corneal thicknesses, this may be translated into a median fraction of transmitted (coherent) light *T*_*coh(stroma)*_ of 51% (interdecile range: 22–83%). Excluding images with central saturation artifact raised our median *T*_*coh(stroma)*_ to 73% (interdecile range: 34–84%). These transparency values are slightly lower than those previously reported, which we attribute to the detection configuration of SD-OCT with a relatively small and selective acceptance angle. No statistically significant correlation between transparency and age or thickness was found. In conclusion, our algorithm provides robust and quantitative measurements of corneal transparency from standard SD-OCT images with sufficient quality (such as ‘Line’ and ‘CrossLine’ B-scan modes without central saturation artifact) and addresses the demand for such an objective means in the clinical setting.

## Introduction

The clinical evaluation of corneal transparency is extremely limited and usually consists of a qualitative and undetailed inspection of visible opacities using a slit-lamp biomicroscope, sometimes with comparison against an arbitrary and subjective grading scale (e.g., 0 to 4 or 5) [1, 2]. Consequently, results are observer-dependent, difficult to standardize or follow, and lack reproducibility. There is a clinical need for an objective and reliable method that enables quantitative corneal transparency assessment, including monitoring capability, towards effective prevention, diagnosis, and treatment of various pathologies.

Several approaches have been proposed to quantify and/or objectively assess corneal transparency [3] or its loss–commonly referred to as “haze”–(e.g., via slit-lamp biomicroscopy [3, 4], the Scheimpflug principle [5, 6], confocal microscopy [7], and optical coherence tomography (OCT) [8]), each one having its own advantages and disadvantages. Among those, so-called corneal densitometry by means of Scheimpflug imaging has gained most widespread usage [9], however this haze analysis is solely based on the measurement of an average grayscale level of a selected area and does not relate to actual tissue properties. In addition, assessment protocols are not standardized, making a direct comparison between devices not feasible [10].

Our team previously developed an objective method for deriving quantitative corneal transparency parameters, directly related to the physics of light propagation in tissues, from depth-resolved optical data (e.g., the Birge ratio as a measure of tissue heterogeneity and the photon mean-free path quantifying the scattering extent in homogeneous tissues); it was validated *in vitro* using high-resolution three-dimensional data obtained by full-field optical coherence tomography (FF-OCT) [11].

The present study translates this work into clinical practice by applying our method to standard two-dimensional cross-sectional images (B-scans) acquired with a clinical spectral-domain (SD-)OCT device. The specific challenges of this clinical application include the much wider field-of-view (FOV) of these OCT devices and the variations due to the positioning of the patient with respect to the FOV, along with the confocal properties of the system (with a fixed focal plane in contrast to FF-OCT, where the relatively small FOV images are always acquired in the focal plane). Our approach thus pays particular attention to the detection and compensation of common instrument-specific acquisition artifacts and employs an automated pre-processing algorithm that includes the computation of a correction mask to standardize the analysis and extraction of tissue-related transparency measures.

## Materials and methods

### Instrumentation and image acquisition

2D OCT (B-scan) images from a standard clinical SD-OCT device (RTVue-XR Avanti OCT; Optovue Inc., Fremont, CA, USA) were used in this study. The SD-OCT device included the cornea-anterior module lens (CAM), specifically the wide-angle lens (CAM-L) configuration (allowing for essentially telecentric scanning across the cornea). Acquisition rates were 26,000 A-scans/second, with 256 to 1024 A-scans/frame. The SLD was centered at 840±10 nm with a spectral bandwidth Δλ = 50 nm (FWHM); light exposure at the pupil was 750 μW. The axial and transverse (theoretical) resolutions in tissue were 5 μm and 15 μm, respectively. The axial image pixel size was calibrated using the automated central pachymetry (= central corneal thickness, CCT) measurements provided by the Optovue software (version 2018.1.1.63), resulting in a 4.3 μm *z*-pixel size. The output images of the clinical device were stored as 8-bit grayscale JPEG files and anonymized after manual USB export. Four acquisition modes were considered (‘Line’, ‘CrossLine’, ‘Pachymetry’, ‘PachymetryWide’), differentiated by their B-scan dimensions and signal-to-noise ratio (SNR). Both ‘Line’ and ‘CrossLine’ scan modes, intended for immediate cross-sectional visualization, have an 8-mm lateral FOV and a 2.2-mm physical scan width; their output images are the result of 30 averaged B-scans. ‘Pachymetry’ and ‘PachymetryWide’ modes, which acquire radial scans across the corneal surface (8 cross-sectional directions 22.5° from each other), are characterized by their respective lateral FOV of 6 mm and 9 mm and by a 1.8-mm physical scan width; their output images are the result of only a few averaged B-scans (exact number unknow, but much lower than 30 given the noisiness of output images), which is sufficient for their epithelial and corneal thickness mapping purposes. All B-scans are transferred from “optical” to “physical distance space” during the manufacturer’s software image reconstruction by means of a dewarp calculation (Snell’s law).

### Patient selection for creation of a normative database

SD-OCT images of *n* = 83 normal corneas from 43 subjects (aged 31±13 years) were included in this study, which was approved by the Institutional Review Board (Patient Protection Committee, Île-de-France V) and adhered to the tenets of the Declaration of Helsinki. The criterion for study inclusion was registration for, but prior to, refractive surgery at the Quinze-Vingts National Eye Hospital’s anterior segment service in the time frame from 2018 to 2021. The absence of a preoperative OCT exam, a decrease in visual acuity of any origin despite correction, the presence of any ocular pathology, or eventual rejection for refractive surgery for any reason were exclusion criteria. Images were collected retrospectively; however, all patients provided informed oral consent to have their images used in research. Two output images per cornea, corresponding to horizontal (nasal-temporal) section views, were processed (i.e., a total of 166 B-scans): one from cross-sectional views (‘Line’ or ‘CrossLine’ mode) and the other one from pachymetry maps (‘Pachymetry’ or ‘PachymetryWide’ mode).

For comparison and discussion purposes, we also analyzed SD-OCT images from 2 pathological corneas with compromised transparency as per “gold-standard” subjective and qualitative image inspection, following the same institutional and ethics guidelines.

Similarly, to get an idea of the repeatability of our method, 2 normal corneas (from the same adult subject) were measured prospectively, with informed consent, 40 times by the same observer on the same day, 10 times per OCT acquisition mode (namely ‘Cross’, ‘Line’, ‘Pachy’, ‘PachyWide’). Intraclass correlation coefficient estimates (ICC_3,k_ and ICC_3,1_) and their 95% confidence intervals [12] were calculated using the Python programming language (Python Software Foundation, v2.7.4) and the pingouin.intraclass_corr function from the Pingouin statistical package (version 0.3.12) [13], based on a two-way mixed-effects model, in terms of consistency for multiple (ICC_3,k_) and single (ICC_3,1_) measurements, considering the acquisition modes as fixed raters.

Note that for statistical analysis and the presentation of our results, 95% confidence intervals (CI_95_) of normally distributed data were determined according to Student’s t-distribution coefficient *t*; for a given variable *x*, $CI_{95}=\bar{x}\pm t\;SD$ where SD denotes the standard deviation. For samples with size *n*>30, *t* = 1.96. For non-normally distributed data, the interdecile range (IDR; the range between the 10^th^ percentile and the 90^th^ percentile) is provided.

### Pre-processing algorithm

A pre-processing algorithm was developed in Python (v2.7.4) and is available in the following repository: https://github.com/maelle-v/FitOCT-preprocessing [14]. This procedure is performed to correct instrument-dependent artifacts and standardize the output images of the clinical OCT device, before extracting the in-depth attenuation profile from the corneal stroma.

**Fig 1:**
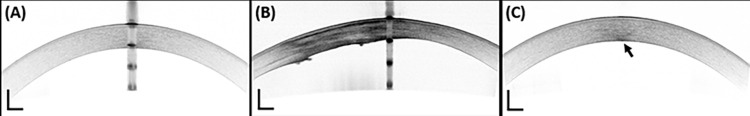
Central artifacts observed in clinical SD-OCT images of corneas (RTVue-XR Avanti OCT; Optovue Inc., Fremont, CA, USA). (A-B, Saturation artifact) Prominent central artifact with a repetitive pattern of back-reflections from the air-tear(-epithelial) interface and endothelium-aqueous interface in the example of a (A) normal cornea and (B) pathological cornea. (C, Posterior stromal artifact) Less prominent central artifact with a hyperreflective region in the posterior stroma (arrow). Scale bar lengths: 500 μm.

We differentiate between two “hyperreflective” artifacts, observed centrally, which are associated with the instrumental configuration and patient positioning and are often termed central artifacts [15]. (1) A prominent central artifact, affecting the entire apex-centered column of the image (that we term saturation artifact; Fig 1A and 1B), with strong specular back-reflection of the incident light at the air-tear(-epithelial) interface (as well as the endothelial-aqueous interface), which saturates the line camera of the SD-OCT’s spectrometer and results in a hyporeflective region between the corneal surfaces. The artifact’s periodic pattern (not observed with swept-source OCT systems) is due to the harmonics generated by the Fourier transform (employed as part of the SD-OCT signal reconstruction) of the sharp-edged shape of the saturated interferograms [16]. (2) A less prominent central artifact (Fig 1C), with a hyperreflective region in the posterior stroma (that we term posterior stromal artifact; arrow in Fig 1C; see Discussion for further details).

**Fig 2:**
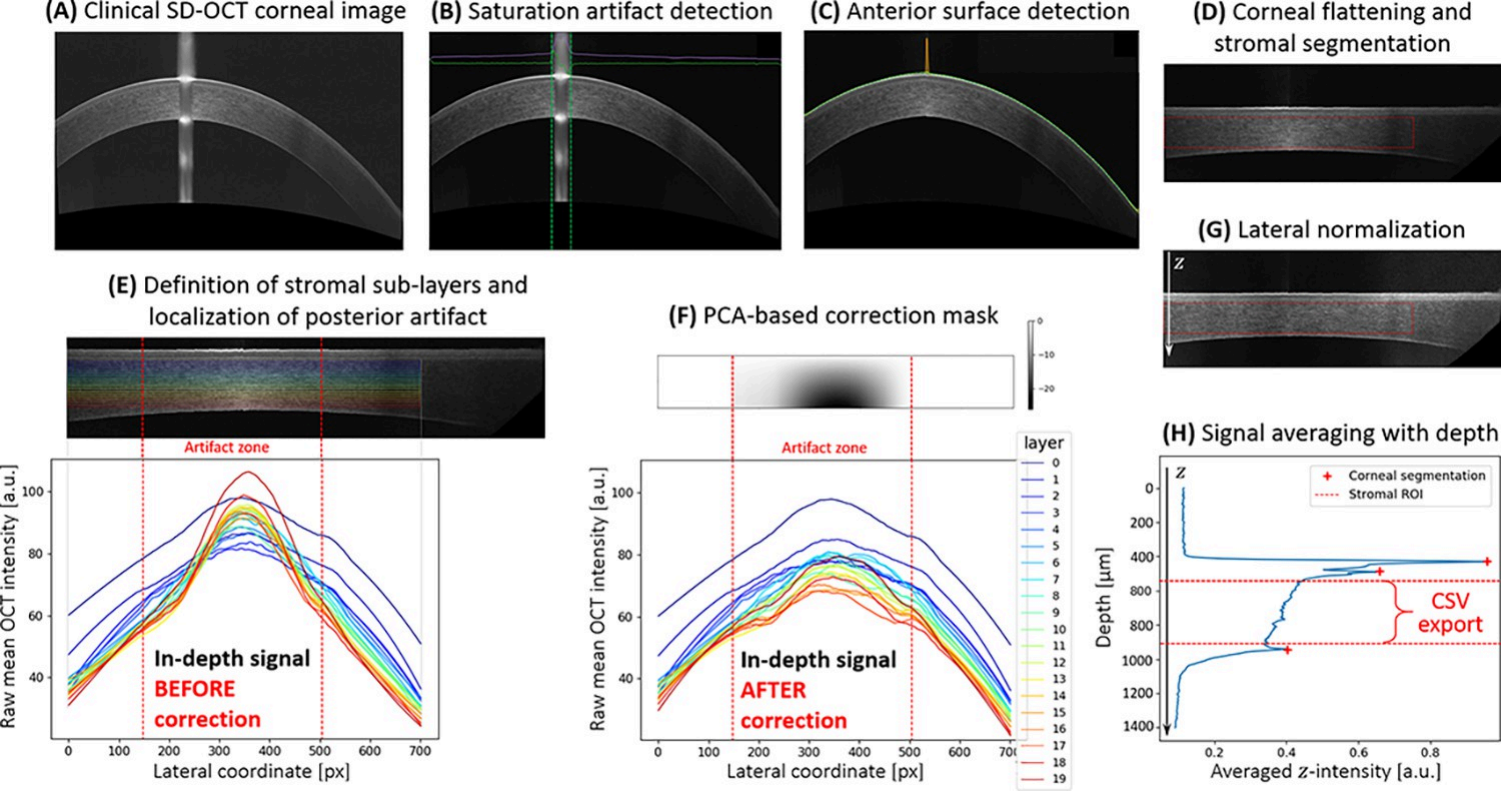
Graphical representation of the pre-processing algorithm with individual steps for SD-OCT corneal image standardization and stromal in-depth intensity profile extraction. (A) Output image of the clinical SD-OCT device (‘Line’ scan mode). (B) Saturation artifact detection after histogram sliding to adjust exposure. (C) Detection of anterior surface. (D) Numerical flattening of the cornea and segmentation of the apex-centered stromal region of interest (ROI). (E, F) Computation of a customized correction mask to account for the posterior stromal artifact. (G) Lateral normalization of the flattened and corrected image. (H) Extraction of the stromal in-depth intensity profile.

The automated steps of the pre-processing algorithm, dealing numerically with those acquisition artifacts, are illustrated in Fig 2. Each step, associated with a sub-figure, is summarized below (and detailed in the associated GitHub page [14]:

- **Clinical OCT image import and optional exposure restoration.** The signal-to-noise ratio of the image (SNR_2D_) is computed.
- **Detection and optional removal of the saturation artifact** (see Fig 1A, 1B). A derivative approach enables automated detection (or not) of the apex-centered saturation artifact; if detected, the columns associated with the saturated area are cropped from the OCT image. User consent is required; if denied, a cursor enables manual (x-) segmentation of this artifact.
- **Detection of anterior surface**. The local maxima corresponding to the depth (z-) coordinate of the air-tear film interface are determined based on a SNR_2D_-dependent thresholding procedure.
- **Flattening of the cornea and stromal segmentation**. Each column of the image is axially translated so that all anterior surface z-coordinates (located in step C) horizontally match. **The region of interest (ROI) delineates stromal boundaries**; it is determined for an apex-centered 6-mm-wide lateral area (x-axis), directly overlying the eye’s pupil and hence most relevant to vision, and lies between the epithelial basement membrane (EBM) and the endothelium (z-axis), located via peak detection, ± two axial (z-) margins: a 50-μm margin after the epithelium-EBM/Bowman’s layer interface as well as a 30-μm margin before the endothelium-aqueous interface. Those margins, chosen empirically, assure not only exclusion of both Bowman’s and Descemet’s layers contribution, respectively, but also avoid contributions of any inhomogeneous portions present in normal stromal regions (e.g., in the anterior 10% of the stroma where keratocyte density is highest [17]). User consent is required; if denied, a cursor enables manual segmentation of the stromal ROI.
- **Lateral localization of the posterior stromal artifact** (arrow in Fig 1C). This step is based on a principal component analysis (PCA) of the mean lateral (x-) intensity of 20 stromal sub-layers of a constant thickness (Fig 2E) and color-coded according to depth (i.e., the warmer the color, the deeper the sub-layer; see S1 Fig for more details).
- **Computation and application of a customized correction mask to compensate for the posterior stromal artifact.** A second PCA is performed on the same input data in the artifact zone (i.e., the specifically located x-coordinate range); the customized intensity correction mask for the posterior stromal artifact (Fig 2F, top) is derived from the 1^st^ principal component eigenvalues of this second PCA. Upon application of the mask, one can observe in Fig 2F, bottom, that the intensities of the deepest sub-layers have been numerically attenuated (in comparison to Fig 2E, bottom).
- **Normalization of the image to compensate for the peripheral loss of intensity due to the geometry of the corneal curvature.** The corrected image is normalized with respect to the smoothed intensity signal at the anterior surface.
- **Averaging of the final image into a single averaged A-scan to extract the mean OCT intensity as a function of depth.** This stromal in-depth attenuation profile is exported as a CSV file.

The mean computation time for the entire pre-processing algorithm is around 2.3 seconds, excluding the GUI interaction time dedicated to user approval (94-bit OS, Inter® Core™ i7-8665U, CPU at 1.9 GHz and 16 GB DDR4 RAM).

### Fitting algorithm and extraction of quantitative transparency parameters

The averaged stromal backscattering profile is in turn analyzed using our developed Bayesian approach-based algorithm (R Core Team, v3.6.3) [18]), which has been described in detail previously [11] and is available using the following DOI: https://doi.org/10.5281/zenodo.2579915. Briefly, a variety of objective parameters, relevant for light propagation in tissues and transparency assessment, are derived from fitting a mono-exponential decay model to the OCT signal as a function of stromal depth. Among the objective parameters are the **signal-to-noise-ratio (SNR)** of the pre-processed attenuation (stromal z-) profile, the **Birge ratio (*B***_***r***_**)**, equivalent to a reduced *χ*^2^, that quantifies stromal homogeneity (homogeneous if *B*_*r*_~1, inhomogeneous if *B*_*r*_≫1), and the photon or **scattering mean-free path** (**ℓ**_***s***_; a major indicator of scattering extent and thus of transparency of a medium).

Note that in the normal (homogeneous) stroma, the incoming coherent wavefront is exponentially attenuated by scattering processes (following a Lambert-Beer law). The propagation distance corresponding to an attenuation by a factor 1/*e* is called the scattering mean-free path (ℓ_*s*_). Given that the average OCT signal at any given depth is proportional to the intensity of the incident wavefront, its measurement permits the assessment of ℓ_*s*_. ℓ_*s*_ together with the stromal thickness (more specifically, the stromal ROI) permits to calculate the **fraction of the transmitted coherent wavefront** as:

$$T_{coh(stroma)}=exp(-\frac{stromal\;thickness}{l_{s}}).$$

Note that the latter expression may be directly related to Strehl ratio reduction and thus retinal PSF broadening, and as such may be used to create a link with visual function or acuity [11].

With regards to computational cost, a 1000-iteration Bayesian inference takes approximately 2.5 minutes per analyzed image.

## Results

### Image-quality and stromal-homogeneity assessment

**Fig 3:**
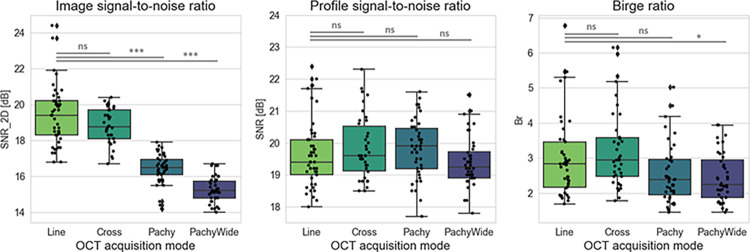
Image quality and stromal homogeneity derived from SD-OCT images: SNR_2D_ of clinical images, SNR of 1D attenuation profiles, and Birge ratio (B_r_) of the exponential fit. The results of pairwise comparisons (with a Tukey HSD posthoc test) are represented above the box plots; “ns” stands for non-significant, while p-values (*P*) lower than 0.05 are considered as significant (* stands for *P*<0.05, ** for *P*<0.01, and *** for *P*<0.001). A total of 166 B-scans were analyzed, of which 45 were Line scans, 38 CrossLine scans (here ‘Cross’), 43 Pachymetry scans (here ‘Pachy’), and 40 PachymetryWide scans (here ‘PachyWide’).

In our sample of *n* = 83 normal corneas, the quality of clinical OCT images depends on the acquisition mode (see also S1 Text, S1 Table, and S2, S3 Figs), as illustrated by the distribution of bidimensional signal-to-noise ratios (SNR_2D_) in Fig 3, left: we observe a significant loss of ~3 dB in pachymetry mapping images (‘Pachy’, ‘PachyWide’) compared to cross-sectional images (‘Line’, ‘Cross’). However, this difference in image quality was found to have no significant impact on the quality (SNR) of the in-depth stromal attenuation profile extracted from the clinical images (mean difference < 0.4 dB; see Fig 3, middle); all extracted profiles can theoretically be analyzed with the fitting algorithm.

Calculated Birge ratios vary slightly with the OCT acquisition mode, but remain rather close to unity (*B*_*r*_<10 see Fig 3, right; IDR_all modes_: 1.8–4.1, IDR_‘Line’&’Cross’_: 1.9–5.1) compared to heterogeneous corneas (*B*_*r*_≫1) [11, 19]. Hence every normal cornea included in our study is considered to have a homogeneous stroma and can be used to establish a representative dataset of normative transparency values via the calculation of ℓ_*s*_.

### Determination of normative transparency values

Even with every available OCT attenuation profile having a sufficient SNR and an acceptable Birge ratio, we established our normative database for corneal transparency measures using only high-quality and high-reliability images acquired in cross-sectional modes, that is, ‘Line’ (*n* = 45) and ‘Cross’ (*n* = 38) OCT scans.

**Fig 4:**
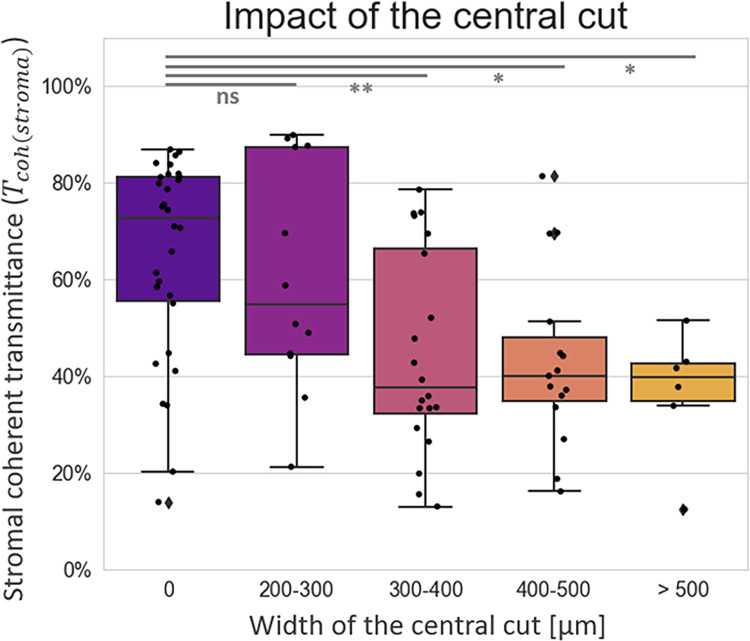
Impact of saturation-artifact removal on the stromal coherent transmittance. The “central cut” corresponds to the part of the image that has been cropped after detection of a saturated area (step B in the pre-processing algorithm, see Fig 2). The central cut width is either null or superior to 200 μm. The results of pairwise comparisons with Tukey HSD posthoc tests are represented above the box plots; “ns” stands for non-significant, while p-values (*P*) lower than 0.05 are considered significant (* stands for *P*<0.05, and ** for *P*<0.01).

We observe an impact of the removal of the saturation artifact on the measured corneal transparency parameters: the wider the saturated zone, the lower *T*_*coh*(*stroma*)_ (Spearman’s rank correlation test between artifact width and *T*_*coh*(*stroma*)_: *ρ* = −0.44, p-value = 10^−5^). This impact is significant for central cuts larger than 300 μm, as illustrated in Fig 4.

**Fig 5:**
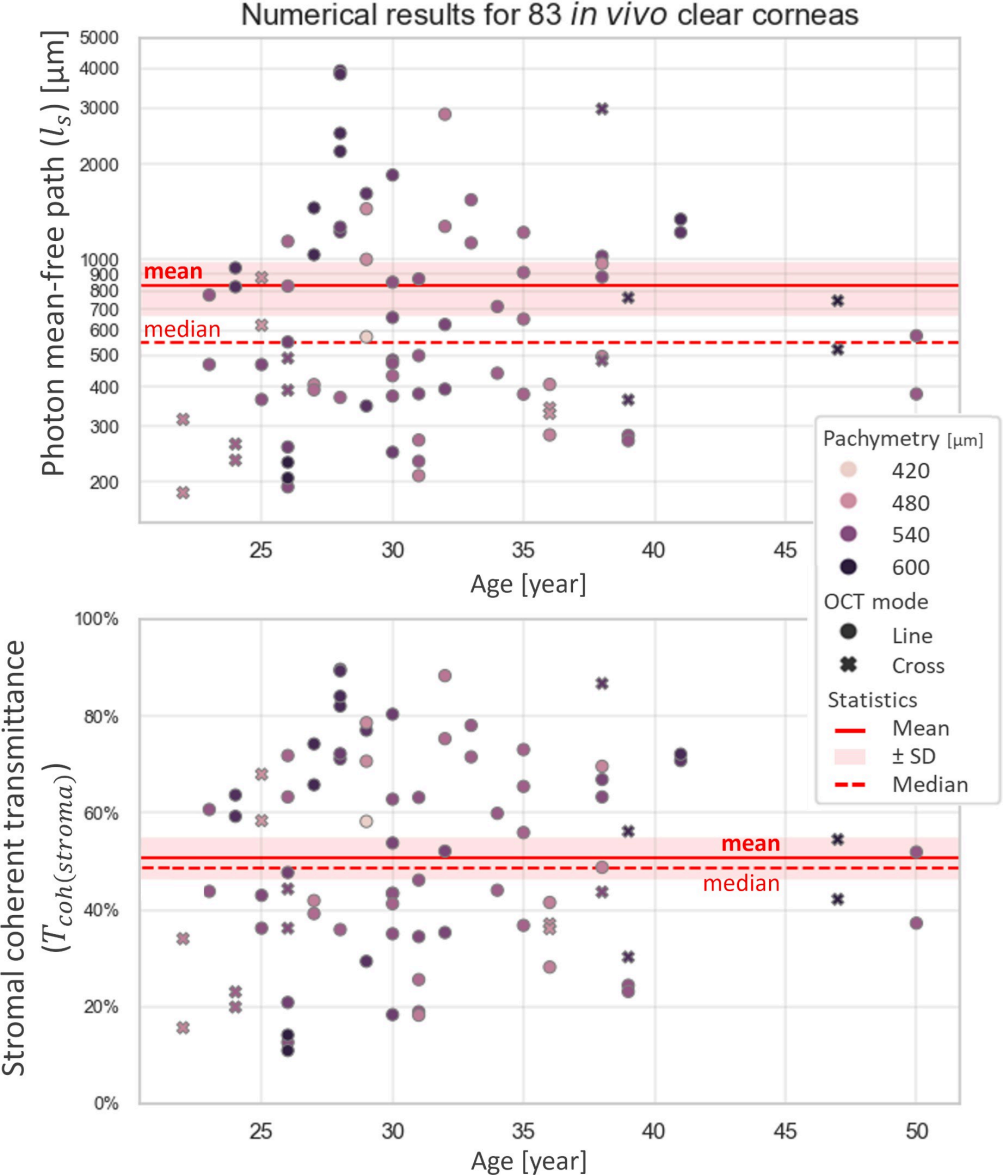
Transparency measures of normal corneas, with subject age as x-axis and central pachymetry (central corneal thickness, CCT) as hue: (top) *ℓ*_*s*_, (bottom) *T*_*coh*(*stroma*)_, (left) *n* = 83 (i.e., all clinical images), (right) *n* = 30 (i.e., clinical images without saturation artifact). Red shades correspond to 1.96 times the standard error (SE = $\mathrm{SD}/\sqrt{n}$) on both sides of the mean. Precision is ±120 μm for *ℓ*_*s*_ measurements and ±9% for *T*_*coh*(*stroma*)_ measurements in ‘Line’ mode, and respectively *Δℓ*_*s*_ = ±230 μm and *ΔT*_*coh*(*stroma*)_ = ±18% in ‘Cross’ mode. No correlation was found between transparency and age or corneal thickness in our group of patients.

Results of the scattering mean-free path and coherent transmittance measurements (both including all corneal images, *n* = 83, and only including those without central artifacts, *n* = 30) are shown in Fig 5 (with more details on the distributions given in S4 Fig and S2 Text). We obtain a lognormal distribution of photon mean-free path values, with median(ℓ_*s*_)= 570 μm, min(ℓ_*s*_) = 190 μm, max(ℓ_*s*_) = 4100 μm, interdecile range (IDR): 270−2400 μm for the entire dataset (*n* = 83), and median(ℓ_*s*_)= 1240 μm, min(ℓ_*s*_)= 230 μm, max(ℓ_*s*_) = 3000 μm, interdecile range (IDR): 400−2500 μm for the reduced dataset without central artifacts (*n* = 30). These translate into a bimodal distribution for the fraction of transmitted coherent light, with median(*T*_*coh*(*stroma*)_) = 51%, min(*T*_*coh*(*stroma*)_) = 13%, max(*T*_*coh*(*stroma*)_) = 90%, IDR: 22−83%, and bimodal peaks around 38% and 80% for *n* = 83, and median(*T*_*coh*(*stroma*)_) = 73%, min(*T*_*coh*(*stroma*)_) = 14%, max(*T*_*coh*(*stroma*)_) = 87%, IDR: 34–84% for *n* = 30.

The corneal thickness in our entire group ranges from 469 μm to 602 μm (CI_95_) and the associated mean percentage of the analyzed stromal depth (i.e. in-depth stromal ROI) corresponds to 74% (SD = 4%) of the full corneal thickness; we observe no significant correlation between coherent transmittance and age or corneal thickness (Spearman’s rank correlation test: *ρ* = 0.10, p-value = 0.39 for age, and *ρ* = 0.17, p-value = 0.12 for corneal thickness, respectively); similarly, no significant correlation between age and corneal thickness is observed (Spearman’s rank correlation test: *ρ* = −0.02, p-value = 0.85). These conclusions remain the same when tests are performed on the reduced sample (*n* = 30) of images without saturation artifact.

Finally, we note that the grouping factor at the subject level (considering *n* = 80 corneas from 40 eye pairs) explains 98% [96–99%] of the total variance of central corneal thickness (CCT) and 55% [30–73%] of the total variance of *T*_*coh*(*stroma*)_ measurements (ICC_1,1_ computation based on a one-way random effects model, for absolute agreement and single measurement: p-values ≪0.05). In other words, two normal corneas from a single person are 98% more likely to have equal thickness and 55% more likely to have similar transparency measures than if they are taken at random. Similarly, there is no significant difference between left and right eyes in CCT or transparency measurements for images with saturation artifact narrower than 300 μm (Wilcoxon’s signed-rank test: W = 13, p-value = 0.86 for CCT and W = 60, p-value = 0.66 for *T*_*coh*(*stroma*)_).

## Discussion

In conclusion, the method described in this article enables the extraction of objective and quantitative corneal transparency parameters, directly related to tissue properties, in routine clinical practice, from standard SD-OCT images. A pre-processing algorithm standardizes image analysis while eliminating common instrument-related artifacts, before an intensity profile along stromal depth is computed; the Birge ratio verifies the model adequacy of a Lambert-Beer law (mono-exponential decay) and thus assesses tissue homogeneity, followed by the photon mean-free path that further quantifies stromal light scattering in homogeneous corneas (i.e., those with low Birge ratios) along with the percentage of transmitted coherent light that associates the impact on visual acuity, contrast sensitivity, and glare.

Assessment of the attenuation coefficient by means of OCT has increasingly been used for characterization of several tissue types [20, 21]. How quickly the signal falls off with depth is the result of both absorption and scattering of incident light inside a medium. However, even in opaque tissues, such as skin, the effect of absorption is negligible at typical near-infrared OCT wavelengths, so that depth-dependent attenuation is mainly an indicator of scattering extent and can hence be used to quantify transparency.

Unlike time-domain (including full-field [11]) OCT systems, the optical components of the sample arm are fixed in SD-OCT, generating a set focal plane within the sample and causing a decrease of sensitivity with increasing distance from the location of focus (so-called confocal function) [22]. In addition, there is a sensitivity fall-off with imaging depth introduced by the frequency sampling limits of the OCT’s spectrometer (so-called frequency “roll-off” [21]). These device-specific “limitations” (i.e., the confocal function and frequency roll-off) affect the depth-dependent OCT signal and create systematic errors or biases that can influence the accuracy of quantitative tissue analysis with OCT [23]. While in the laboratory setting, these parameters can easily be calculated or experimentally determined [24], this is not always feasible with clinical systems, with no ready access to detailed technical specifications or raw data.

To facilitate assessment of tissular backscattering properties by means of “black-box” clinical OCT systems, any potentially limiting device-specific and intrinsic system parameters are taken into account by our approach via a numerical retro-engineering procedure used to eliminate associated biases and standardize image analysis. Aside from SD-OCT’s usual data-processing steps (e.g., FFT, DC subtraction, image compression), we have no evidence of contrast-adjustment methods having been used in the generation of the exported SD-OCT images from the standard clinical device (RTVue-XR Avanti, Optovue Inc.) employed in this study. Indeed, we do observe a mono-exponential decay of OCT intensity in the stroma of normal corneas (as expected in homogeneous media [11]) whereas any non-linear process, including logarithmic contrast enhancement, would have prevented its observation by compromising the linear relationship between the OCT signal and tissular backscattering. In addition, we estimate the frequency roll-off to have a negligible impact on our transparency analysis (which is based on the extraction of attenuation parameters from the averaged stromal OCT signal over a 6-mm-wide ROI) given the limited thickness of the stroma compared to the axial FOV (see S5 Fig). Similarly, the confocal function is estimated to have no major impact in the central 6-mm ROI, especially with the depth of focus being doubled in scattering media, as illustrated in S5 Fig [24]. In any case, systematic image analysis ensured by our pre-processing algorithm accounts for residual signal loss in depth, including in peripheral areas. Our pre-processing algorithm differentiates between two centrally observed “hyperreflective” artifacts, namely a saturation artifact (see Fig 1A and 1B) and a posterior stromal artifact (see Fig 1C). Interestingly, the latter, which is identified and accounted for using a PCA-based approach that includes the computation of a customized correction mask, is not observed in SD-OCT images of a corneal phantom containing spherical shaped scatterers (Cornea model eye, Rowe Technical Design Inc., Dana Point, CA, USA; S6 Fig). This artifact may thus only originate from signal reconstruction in a layered medium (e.g., stromal lamellae in real corneas), and be reinforced slightly by the confocal function of the SD-OCT system as well as by corneal refraction.

Corneal curvature and refraction, affecting the optical path through the cornea, could have been considered in a more complex flattening scheme (e.g., Goode homolosine projection). However, a calculation based on a simple geometric consideration shows that translation of each image column, as employed by our flattening procedure, rather than a flattening following the refracted path, only leads to a maximal error of +0.8% (at the edges of the ROI, ± 3 mm from the corneal apex), which we find acceptable. Any potential residual image distortions after numerical standardization and correction are reflected by the 95% confidence interval of the output parameters (e.g., ±9% for the coherent transmittance of normal corneas imaged with ‘Line’ scan mode).

Corneal curvature also leads to a peripheral loss of intensity by deflecting the acceptance angle of the system. The normalization step of our pre-processing algorithm (see Fig 2G) compensates for any inhomogeneous illumination.

Using medium-resolution cross-sectional images (i.e., ‘Line’ and ‘Cross’ modes of the RTVue-XR Avanti OCT by Optovue Inc. found to have excellent reliability) without saturation artifact from 30 normal corneas, we established normative measures of corneal transparency—based on the scattering mean-free path, ℓ_*s*_ (median = 1250 μm, IDR: 400–2500 μm), in homogeneous stromal ROIs (*B*_*r*_, IDR: 1.9–4.0), notably the fraction of transmitted coherent light, which considers stromal thickness, *T*_*coh*(*stroma*)_ (median = 73%, IDR: 34–84%)—that may serve as further reference. Images from thickness mapping scan modes (e.g., ‘Pachy’, ‘PachyWide’) were found to be inaccurate and should not be used for quantitative signal analysis. Indeed, these images turned out to be a source of fixed biases: their poorer SNR (due to a lower number of averaged B-scans per final image) is associated with an uneven sampling of the in-depth signal notably in the anterior stroma, which in turn may lead to inaccurate fitting in that region. The precision of our measurements on cross-sectional images (i.e., ‘Line’ and ‘CrossLine’) could be statistically improved by analyzing several same-eye OCT images and averaging the associated results, as illustrated in S2 Fig (resulting in dividing the uncertainty of the mean value by the square root of the number of scans), as has been previously done to improve the repeatability of quantitative measurements with the RTVue OCT system by Optovue [25]. For example, by averaging the measures derived from 3 ‘Line’ or ‘Cross’ OCT images, we would attain a ±5% precision for the measurement of stromal coherent transmittance (versus ±9% and ±18% for single measurements in ‘Line’ and ‘Cross’ mode, respectively). This precision could even be further improved to ±3% if analyzing 5 or more images in ‘Line’ or ‘Cross’ mode (see S2 Fig).

**Fig 6:**
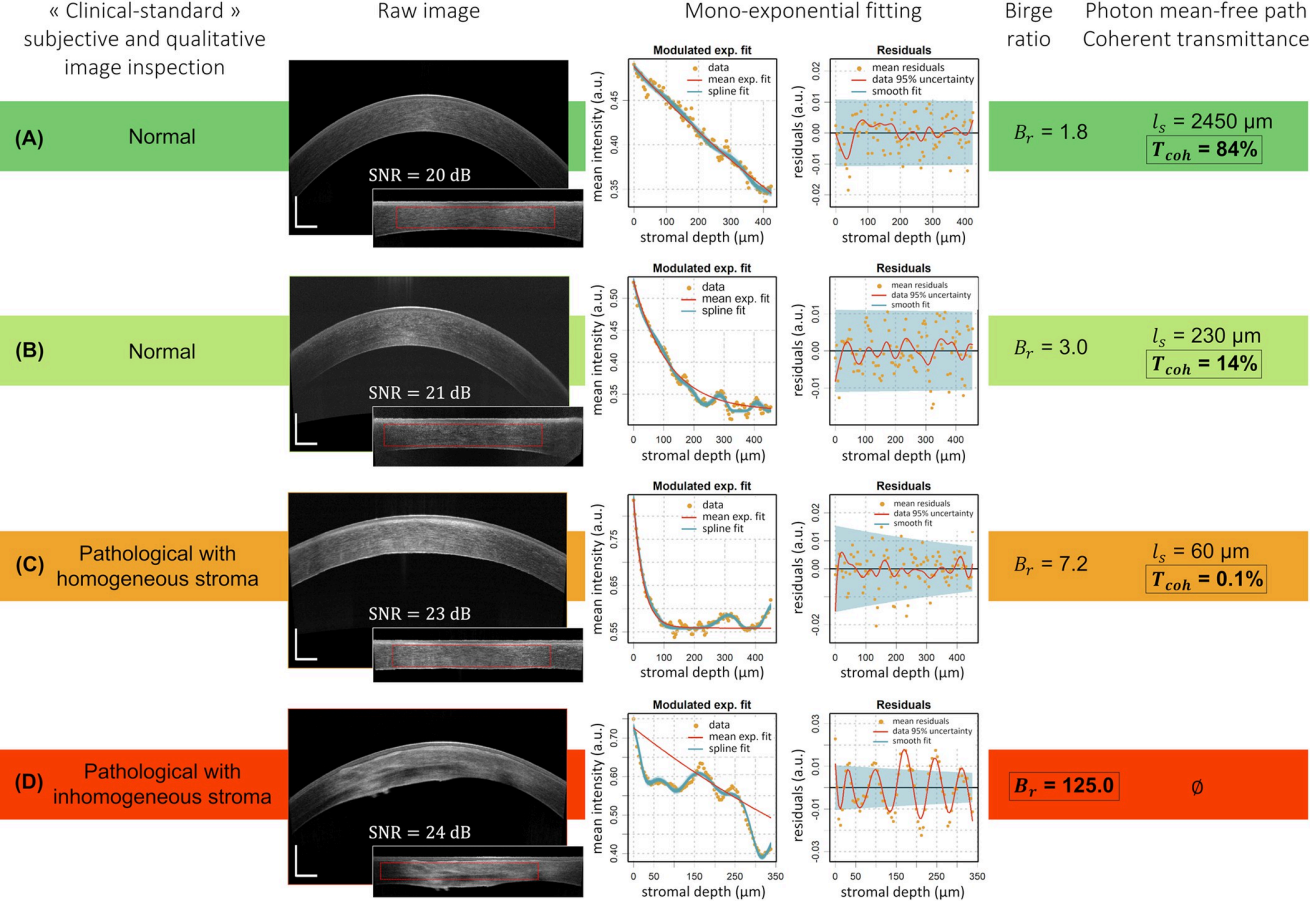
Graphical representation of four representative clinical cases. Each row comprises a SD-OCT cross-sectional ‘Line’ image of a typical *in vivo* human cornea with corresponding flattened and normalized image (the apex-centered ROI appears as a red dotted frame), the associated mean intensity depth profile and mono-exponential fitting analysis (see [11] for further details on fitting analysis). (A, B) Normal corneas (0.0 logMAR visual acuity) from our n = 83 sample with comparable Birge ratios, but (B) shows a faster decaying stromal depth profile and thus has a lower photon mean-free path (*ℓ*_*s*_) and associated fraction of transmitted coherent light (T_coh(stroma)_). (C, D) Pathological corneas with compromised transparency as per “gold-standard” subjective and qualitative image inspection, with (C, Fuchs dystrophy) showing homogeneous scattering in the stroma (B_r_< 10), resulting in a very fast in-depth decay and thus extremely low T_coh(stroma)_, and (D, keratoconus) with visible heterogeneities (very heterogeneous scattering) in the stroma (B_r_≫ 10); transparency assessment via a mono-exponential fitting of the in-depth profile is hence inadequate. Scale bar lengths: 500 μm.

Our values for transmitted coherent light, reflecting stromal transparency (*T*_*coh*(*stroma*)_), are slightly lower than those for full corneal thickness previously reported in the literature at 840 nm or the closest available wavelength (see Table 1). This may be partly explained by the detection configuration of SD-OCT devices, with small numerical apertures that favor depth of field (NA ~ 0.1); because backscatter from the corneal stroma is integrated over 2 times the numerical aperture of the sample arm, this results in a relatively small and selective acceptance (solid) angle on the order of 10^−2^ steradian (*sr*). Our coherent stromal transmittance measures are therefore smaller than those obtained with methods using a detector that is subtended by a larger solid angle, even for direct measurements (e.g., made with an integrating sphere). Indeed, the mean fraction of transmitted coherent light that we obtained for our group of normal corneas is well compatible with literature values that were obtained with measurement methods whose detection (solid angle) was confined to the vicinity of the ballistic propagation direction (such as in [30, 31]). We still observe a high variation of transparency values (cf. IDR). It is regrettable that most of the prior studies mentioned in Table 1 do not provide the standard deviation of their transmission measurements, when averaged on several corneas, for comparison purposes. Regardless, the reported normal transmittance values should be considered with respect to the solid angle used. A preliminary comparison between healthy subjects and patients, shown in Fig 6, suggests that the *T*_*coh*_ threshold between transparent and scattering corneas may be very low, likely around a few percent. Consequently, a large variation in transparency values above this threshold would only reflect the ‘normal’ range of corneal transmission with respect to coherent light. Similarly, a large variation in corneal haze measures as determined by Scheimpflug densitometry were found in a study involving 588 clinically normal corneas [32].

**Table 1:** Comparison with previously reported measurements of corneal transmittance, at 840 nm or the closest available wavelength [26–31]. Standard deviations for averaged transmittance values are given when available.

Year	Reference	Set-up	Wavelength (nm)	Solid angle (sr)	Measurement [sample size]	Corneal transmittance	Age (yr) Mean [range]
1962	Boettner & Wolter [26]	*in vitro*	840	10^1^ (total)*	Average [6 of 9]	95%	35 [0.3–75]
				10^−4^ (direct)^†^	Single [best observed]	80%	4.5
				10^−4^ (direct)^†^	Single [near the av. of 8]	67%	53
1984	Lerman [27]	*in vitro*	750	—	Single	90%	8
				—	Single	77%	24
				—	Single	68%	80
1990	Beems & van Best [28]	*in vitro*	700	— ^‡^	Average [8]	96% SD ≤ 9%	61 [22–87]
1994	van den Berg & Tan [29]	*in vivo*	700	10^1^ (total)*	Average [10]	94%	51 [14–75]
				10^−4^ (direct)^†^	Average [10]	89%	51 [14–75]
2010	Peyrot *et al*. [30]	*in vitro*	840	10^−7^ (direct)	Single (deswollen cornea)	67%	—
2013	Crotti *et al*. [31]	*in vitro*	840	10^−7^ (direct)	Single (mildly edematous cornea)	48%	—
2021	Present study	*in vivo*	840	~ 0	Average [83]	51%^§^ IDR: [22–83]%	31 [22–50]
					Average [30]^||^	73%^§^ IDR: [34–84]%	32 [24–39]

* Solid angle measured for an integrating sphere with an acceptance angle close to 180° (Ω = 2π[1-cos(170/2)] = 5.7 sr ≈ 101 sr).

† Solid angle measured for an acceptance angle of 1° (Ω = 2π[1-cos(1/2)] ≈ 10–4 sr).

‡ The acceptance angle was not specified. They used a 2.7 mm wide square photodiode implanted in the anterior chamber (if the distance between the front of this photodiode and the center of the cornea was 1.5 mm, the acceptance angle and the solid angle were around 80 degrees and 100 sr, respectively).

§ Median coherent transmittance of the corneal stroma (the stromal ROI is defined in the present article). IDR stands for interdecile range, i.e, the range between the 10th percentile and the 90th percentile.

|| This reduced sample (n = 30 corneas) corresponds to OCT images with no saturation artifact.

We found no significant correlation between age and central pachymetry (central corneal thickness, CCT) in our group of patients (that is relatively young due to the inclusion criteria having been registration for refractive surgery; age range from 22 to 50 years), as was previously reported for subjects younger than 50 years [33]. We also found no significant correlation between age and stromal coherent transmittance (*T*_*coh*(*stroma*)_) at 840 nm, which is in agreement with prior full thickness transmittance measures [26–29] (note that a decrease in corneal light transmission with age was reported in [27] but later contradicted in [29]). Similarly, no age-related differences or correlations were previously found for corneal birefringence (which, as corneal transparency, is related to stromal structure) [34]. Further work will include a larger group of patients (including with various pathologies) with broader age ranges, enabling us to investigate the impact of age on *in vivo* near-infrared stromal transparency in subjects older than 50 years.

We did, however, find an impact of the grouping factor at the subject level (i.e., corneas belonging to the same subject) for central corneal thickness (CCT) and stromal transmittance measurements: it explains 98% [96–99%] of the total variance of CCT (as has been previously described in [35]) and 55% [30–73%] of the total variance of *T*_*coh*(*stroma*)_ measurements. The good agreement between fellow eyes demonstrates the accuracy of the measurements. The residual variance in *T*_*coh*(*stroma*)_ may be explained by intra-subject variations, depending on each eye’s medical history, by the impact of saturation artifacts, as well as by the uncertainty related to single measurements. We noticed no significant difference in CCT or stromal transmittance between right and left corneas of the same subject, as reported for CCT in [33] and for corneal birefringence in [34].

While subjective and qualitative image inspection (and/or grading scale) routinely used by ophthalmologists in clinical practice is capable of distinguishing heterogeneous (abnormal; *B*_*r*_≫1) corneas from homogeneous corneas (*B*_*r*_~1), it may fail to differentiate between those homogeneous corneas with compromised transparency (highly scattering; ℓ_*s*_ < corneal thickness) and those being transparent (ℓ_*s*_≫ corneal thickness, as can be distinguished and classified by our algorithm). This is illustrated in Fig 6 comparing four representative clinical cases (two healthy subjects and two patients), by associating clinical SD-OCT images with their respective (z-) attenuation profiles and objective parameters. The low transparency measurements obtained for some normal corneas (*T*_*coh*(*stroma*)_ between 13% and 30%; e.g. Fig 6B) raise the question of how a decrease in transmitted coherent light impacts patients’ vision from a psychophysical point of view, since those low-coherent-transmittance corneas are still considered “clinically normal” and subjects all have a best-corrected visual acuity of 20/20 (0.0 logMAR) or better. By creating a “normative” database of our parameters for pathological corneas, we shall be able to define thresholds and ranges of these physically relevant parameters in relation to clinically relevant indications, to provide a user-friendly and quantitative tool to ophthalmologists.

Future work will hence focus on the application and optimization of our approach to patients affected by specific corneal pathologies, including but not limited to Fuchs dystrophy, along with the study of the progression of the disease and post-operative follow-up. The computational cost of our algorithm can be improved, notably by a substantial reduction of Bayesian iterations, to facilitate its integration within routine clinical practice and provide an automated assessment of corneal transparency from standard SD-OCT cross-sectional views within a few seconds.

## Supporting information

S1 FigPrincipal component analysis (PCA) of sub-layer mean intensities.(**A**) The PCA input data is expressed using a matrix formalism, each line being the averaged signal at a given depth in the stroma. The input matrix size is *K*×*N* with *K* being the number of sub-layers (here *K* = 20) and *N* the image width in pixels. A first PCA of this input matrix is performed using Python’s sklearn.decomposition.PCA function to define the artifact (x-) coordinates. The *PCA* function is based on a singular value decomposition of the data which projects it to a lower dimensional space; the input data is centered but not scaled. The corresponding eigenvalues and eigenvectors are stored in two matrices, with columns ranked in descending order of component variance, each column being representative of a principal component (PC; axes of the new basis defined by the PCA). (**B**) The first 5 PC eigenvectors (i.e., columns 1 to 5 of the eigenvector matrix) are plotted (left), depicting input data tendencies associated with each of these PCs. The three plots on the right illustrate the non-centered reconstruction of data derived from the corresponding PC (with the same color code as input sub-layers); they are helpful for user interpretation of PCA results. For example, the reconstructed contribution of PC_1_ for sub-layer No. 1 equals the product of $\lambda_{CP_{1,layer1}}$ and the 1^st^ column of eigenvectors matrix, ${\left[I_{PC_{1},x_{0}}\;\cdots \;I_{PC_{1},x_{max}}\right]}^{T}$. The percentage on each figure is the amount of data variance explained by the PC. Considering the robust trend observed in the PC reconstructed data, we obtained the lateral (x-) coordinates of the artifact zone from the two local minima around the central region of the non-centered reconstructed data derived from PC2. A second PCA is performed on the same input data restricted to the artifact zone (i.e., the x-coordinate range defined in the abovementioned step). (**C**) shows the analyzed region (left), while the three plots on the right illustrate the non-centered reconstruction of data derived from the corresponding PC (of the 2^nd^ PCA). The intensity corresponding to a customized correction mask for the posterior stromal artifact is calculated from the reconstructed data derived from the PC1 of this 2^nd^ PCA.(TIF)

S2 Fig Bland-Altman comparison of ‘Cross’, ‘Pachy’, ‘PachyWide’ and ‘Line’ OCT acquisition modes for measures of transmitted coherent light (*T*_*coh*(*stroma*)_). ‘Line’ mode is used as a reference. Top three graphs correspond to right eye (OD) results, bottom three graphs to left eye (OS) results. The graphs show a fixed bias of ‘Pachy’ mode, being +40% for OD and +7% for OS. A multiple comparison of means with Tukey HSD post-hoc test reveals that the +40% bias is significant with ‘Line’ mode considered as a reference.
(TIF)

S3 Fig Convergence of the sample mean *T*_*coh*(*stroma*)_ according to the sample size. The sample size corresponds to the number of analyzed images from the same eye, acquired at the same moment by the same observer. The graphs depict the data for the left eye (OS; left panel) and right eye (OD; right panel) tested for reliability.
(TIF)

S4 FigDistribution of transparency measures on normal corneas, respectively the photon mean-free path (ℓ_*s*_) and the fraction of transmitted coherent light (*T*_*coh*(*stroma*)_). (A, B) Transparency measures of the entire sample (n = 83, ‘Line’ and ‘Cross’ images). (C, D) Transparency measures of the reduced sample (n = 42, ‘Line’ and ‘Cross’ images without saturation artifact or with a saturation artifact narrower than 300 μm). Graphs display the kernel density estimation (KDE) of the data, with respective bandwidth of 100 *μ*m and 5% for *l*_*s*_ and *T*_*coh*(*stroma*)_.The mean values of *l*_*s*_ distributions are computed as $exp(\bar{log(l_{s})})$.(TIF)

S5 FigStromal area of interest and SD-OCT depth of focus illustrated on a clinical image of a normal cornea.The stromal area of interest used in our analysis is highlighted in purple. The horizontal (bold) dashed lines illustrate the extent of the depth of field (i.e., *b* = *αz*_*R*_ with *z*_*R*_ being the Rayleigh range of a Gaussian beam, i.e. the confocal function extent, and *α* = 2 characterizing diffuse backscattering) with a possible location of the focal plane *z*_*f*_ of the system (solid line; arbitrarily chosen for illustration). The value *b* = 850 μm was obtained after calculation based on the known device specifications ($z_{R}=\pi \omega_{0}^{2}/\lambda_{0}$ considering the waist *ω*_0_ as the 15-μm lateral resolution at *λ*_0_ = 840±10 nm). The horizontal (non-bold) dashed lines represent the doubling of the depth of field in scattering media, which applies for OCT imaging under the assumption of simple scattering [24]. Note that the peripheral zones of the cornea are out of focus, which may partly explain the lower signal-to-noise ratio and darkening in those areas; the impact of the SD-OCT’s confocal parameters may not be negligible outside the 6-mm-wide region of interest.(TIF)

S6 Fig SD-OCT image of a corneal phantom (Cornea model eye, Rowe Technical Design Inc., Dana Point, CA, USA). Scale bar lengths: 500 μm.
(TIF)

S1 Text Reliability and precision of the extracted parameters. S1 Table provides intraclass correlation coefficient estimates (ICC_3,k_ and ICC_3,1_) and their 95% confidence intervals that were calculated using Python software version 2.7.4 (Python Software Foundation) and the pingouin.intraclass_corr function from the Pingouin statistical package (version 0.3.12), based on a two-way mixed-effects model, in terms of consistency for multiple (ICC_3,k_) and single (ICC_3,1_) measurements, treating the OCT acquisition modes as fixed raters. Bland-Altman diagrams are provided in S2 Fig for the comparison of inter-raters fixed bias of the average transmitted coherent light, T_coh(stroma)_, with ‘Line’ mode used as a reference. The precision of the extracted parameters as a function of number of measurements is illustrated in S3 Fig.
(DOCX)

S2 Text Shapiro-Wilk normality test results. Results for the entire sample (n = 83, all ‘Line’ and ‘Cross’ images) and for the reduced sample (n = 42, images without saturation artifact or with a saturation artifact narrower than 300 μm) are given.
(DOCX)

S1 Table Reliability measurements of objective parameters. Intraclass correlation coefficient estimates (ICC_3,k_ and ICC_3,1_) and their 95% confidence intervals (CI) are shown, with *k* representing the number of measurements.
(DOCX)
