# Supplementary figures and images for: A new method for in vivo assessment of corneal transparency using spectral-domain OCT
Source: PLoS One. 2023 Oct 5;18(10):e0291613. doi: 10.1371/journal.pone.0291613 (PMC10553212; doi:10.1371/journal.pone.0291613)

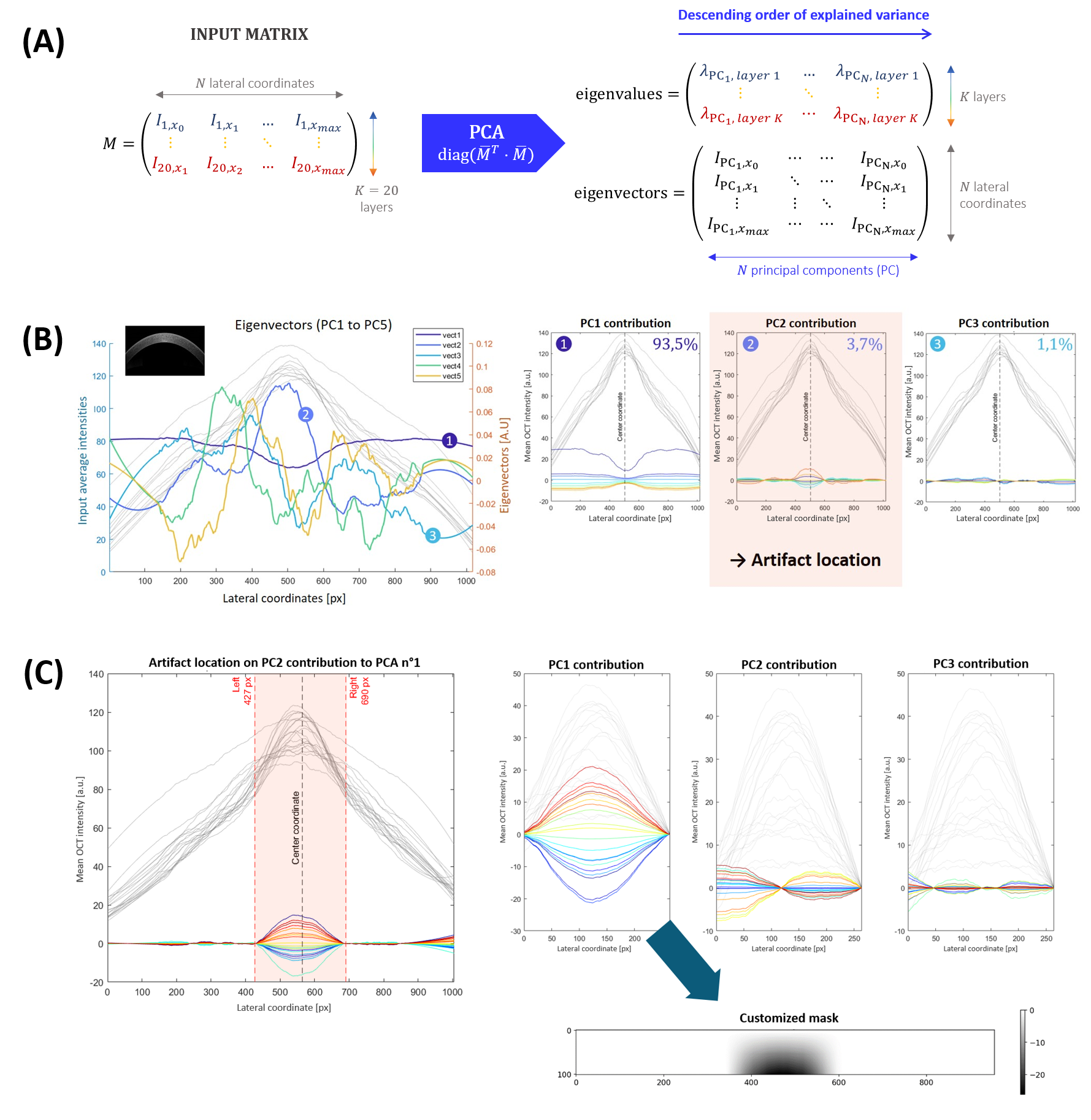

Supplement: S1 Fig — (A) The PCA input data is expressed using a matrix formalism, each line being the averaged signal at a given depth in the stroma. The input matrix size is K×N with K being the number of sub-layers (here K = 20) and N the image width in pixels. A first PCA of this input matrix is performed using Python’s sklearn.decomposition.PCA function to define the artifact (x-) coordinates. The PCA function is based on a singular value decomposition of the data which projects it to a lower dimensional space; the input data is centered but not scaled. The corresponding eigenvalues and eigenvectors are stored in two matrices, with columns ranked in descending order of component variance, each column being representative of a principal component (PC; axes of the new basis defined by the PCA). (B) The first 5 PC eigenvectors (i.e., columns 1 to 5 of the eigenvector matrix) are plotted (left), depicting input data tendencies associated with each of these PCs. The three plots on the right illustrate the non-centered reconstruction of data derived from the corresponding PC (with the same color code as input sub-layers); they are helpful for user interpretation of PCA results. For example, the reconstructed contribution of PC1 for sub-layer No. 1 equals the product of λCP1,layer1 and the 1st column of eigenvectors matrix, [IPC1,x0⋯IPC1,xmax]T. The percentage on each figure is the amount of data variance explained by the PC. Considering the robust trend observed in the PC reconstructed data, we obtained the lateral (x-) coordinates of the artifact zone from the two local minima around the central region of the non-centered reconstructed data derived from PC2. A second PCA is performed on the same input data restricted to the artifact zone (i.e., the x-coordinate range defined in the abovementioned step). (C) shows the analyzed region (left), while the three plots on the right illustrate the non-centered reconstruction of data derived from the corresponding PC (of the 2nd PCA). The intens [file pone.0291613.s001.tif]

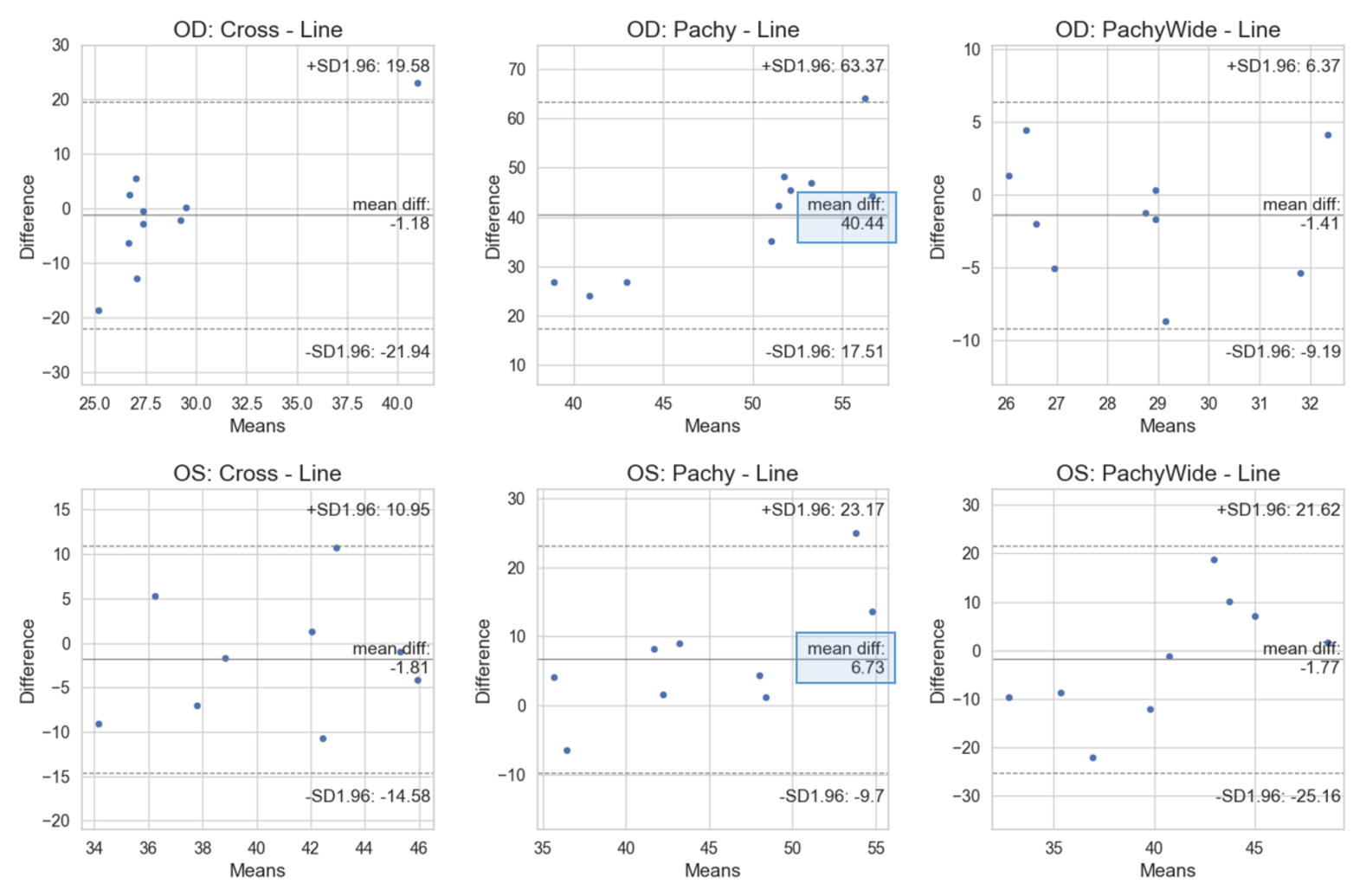

Supplement: S2 Fig — ‘Line’ mode is used as a reference. Top three graphs correspond to right eye (OD) results, bottom three graphs to left eye (OS) results. The graphs show a fixed bias of ‘Pachy’ mode, being +40% for OD and +7% for OS. A multiple comparison of means with Tukey HSD post-hoc test reveals that the +40% bias is significant with ‘Line’ mode considered as a reference. (TIF) [file pone.0291613.s002.tif]

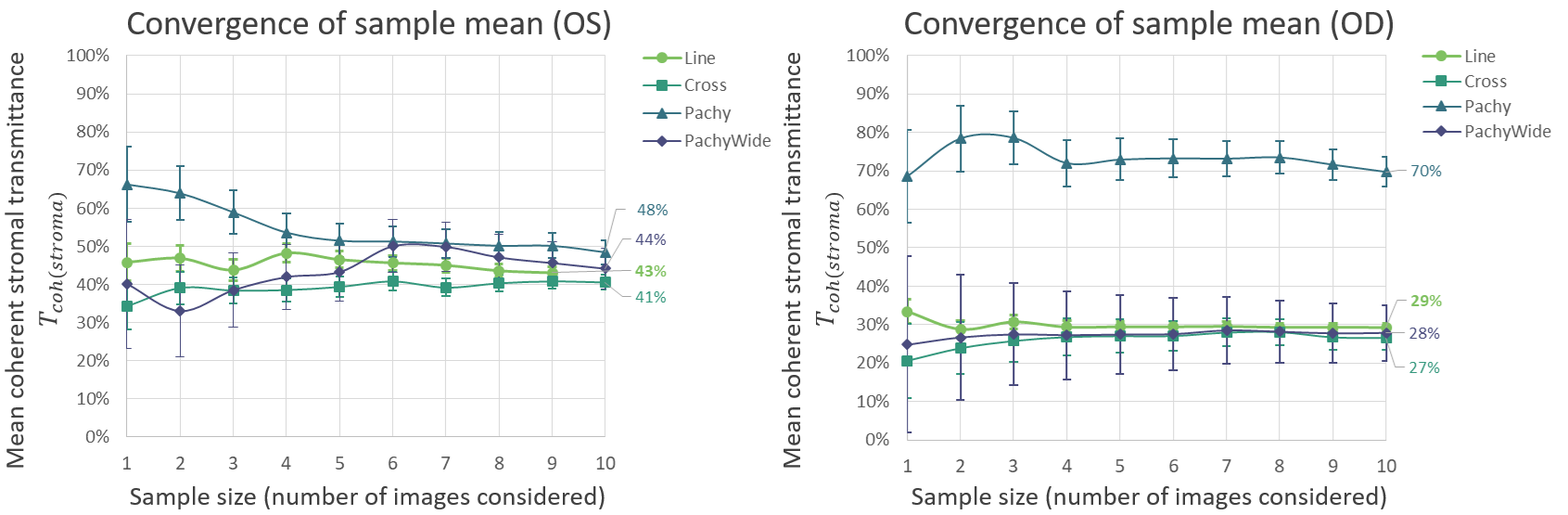

Supplement: S3 Fig — The sample size corresponds to the number of analyzed images from the same eye, acquired at the same moment by the same observer. The graphs depict the data for the left eye (OS; left panel) and right eye (OD; right panel) tested for reliability. (TIF) [file pone.0291613.s003.tif]

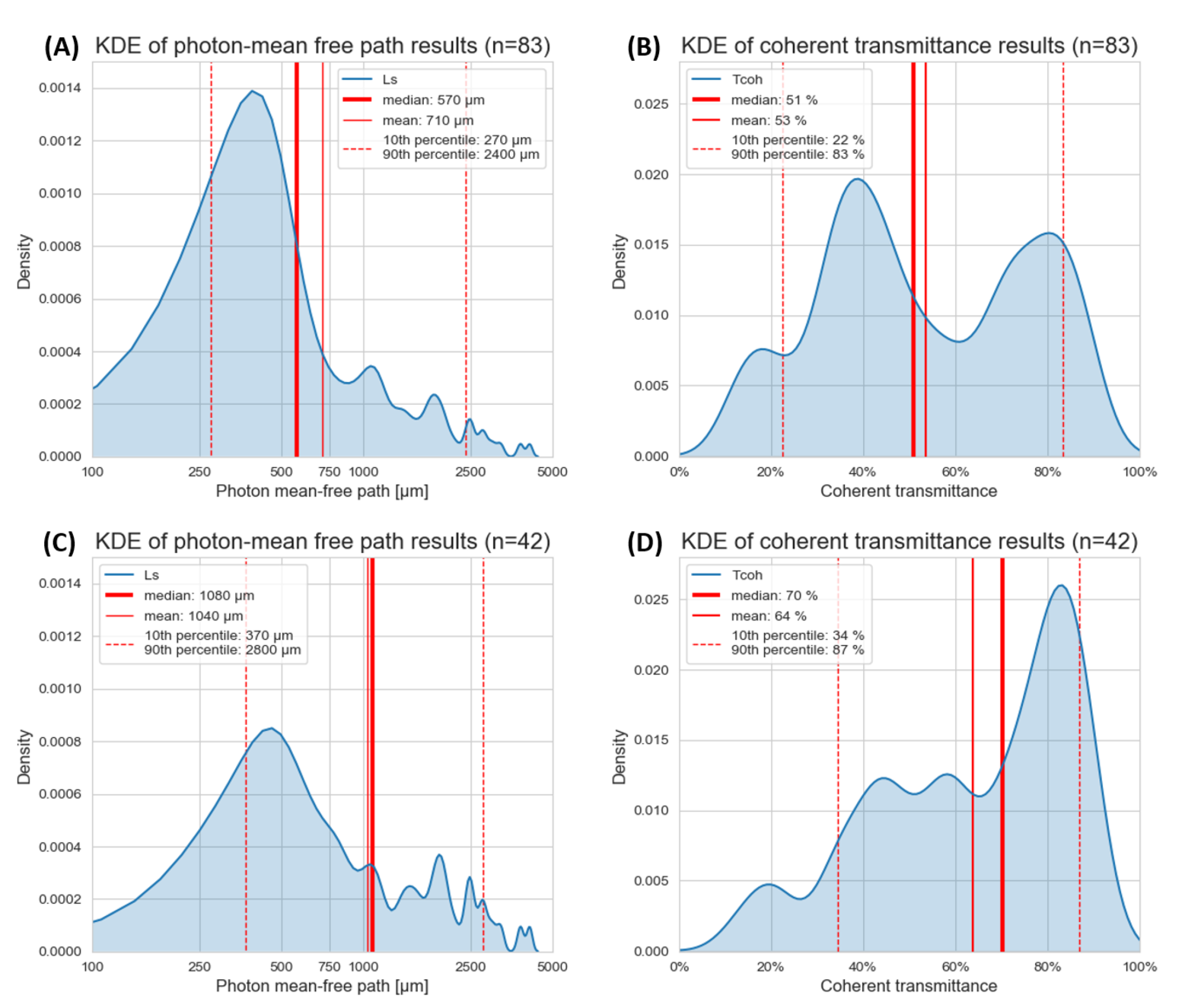

Supplement: S4 Fig — The mean values of ls distributions are computed as exp(log(ls)¯). (TIF) [file pone.0291613.s004.tif]

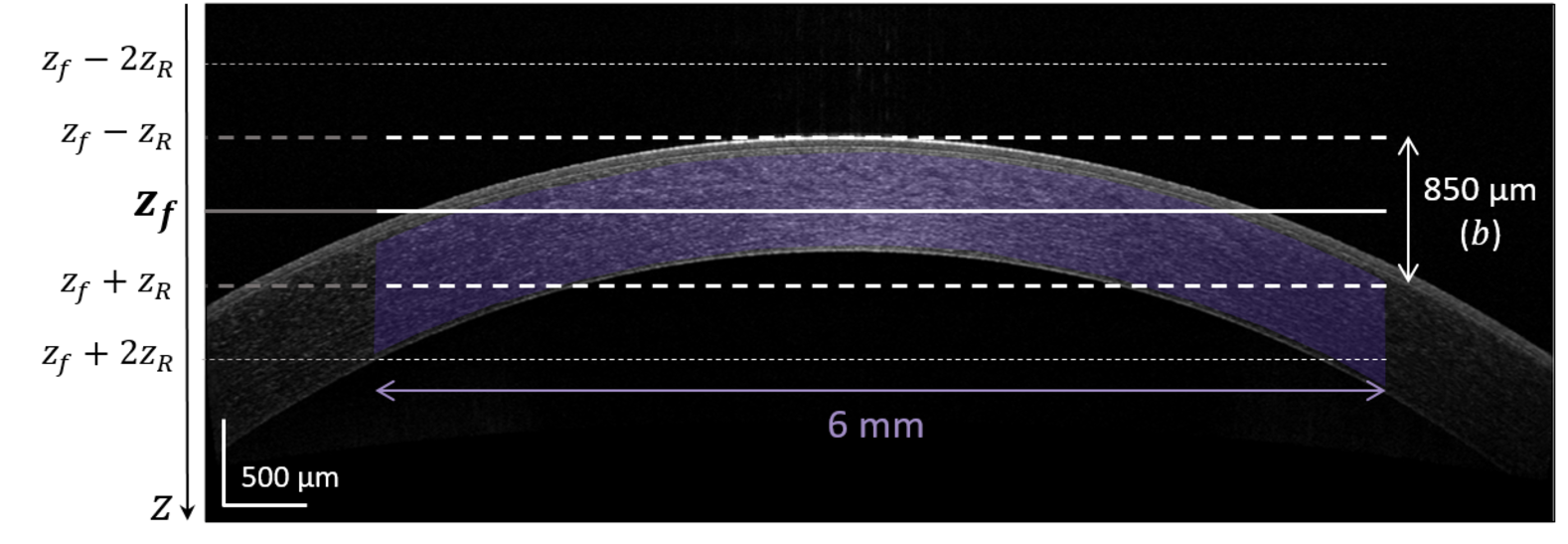

Supplement: S5 Fig — The stromal area of interest used in our analysis is highlighted in purple. The horizontal (bold) dashed lines illustrate the extent of the depth of field (i.e., b = αzR with zR being the Rayleigh range of a Gaussian beam, i.e. the confocal function extent, and α = 2 characterizing diffuse backscattering) with a possible location of the focal plane zf of the system (solid line; arbitrarily chosen for illustration). The value b = 850 μm was obtained after calculation based on the known device specifications (zR=πω02/λ0 considering the waist ω0 as the 15-μm lateral resolution at λ0 = 840±10 nm). The horizontal (non-bold) dashed lines represent the doubling of the depth of field in scattering media, which applies for OCT imaging under the assumption of simple scattering [24]. Note that the peripheral zones of the cornea are out of focus, which may partly explain the lower signal-to-noise ratio and darkening in those areas; the impact of the SD-OCT’s confocal parameters may not be negligible outside the 6-mm-wide region of interest. (TIF) [file pone.0291613.s005.tif]

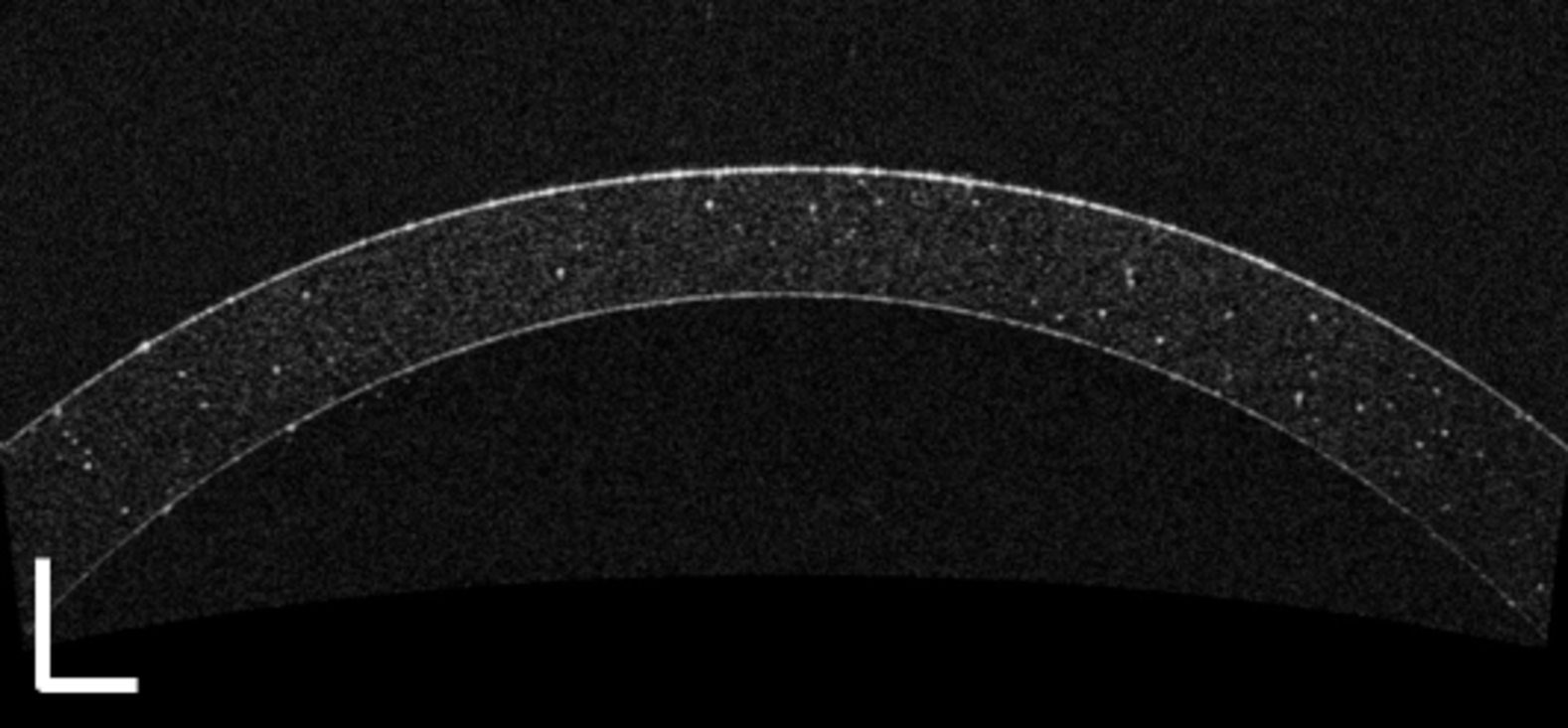

Supplement: S6 Fig — Scale bar lengths: 500 μm. (TIF) [file pone.0291613.s006.tif]
