# Supplementary material for: A new method for in vivo assessment of corneal transparency using spectral-domain OCT
Source: PLoS One. 2023 Oct 5;18(10):e0291613. doi: 10.1371/journal.pone.0291613 (PMC10553212; doi:10.1371/journal.pone.0291613)
Supplement: S1 Text — S1 Table provides intraclass correlation coefficient estimates (ICC3,k and ICC3,1) and their 95% confidence intervals that were calculated using Python software version 2.7.4 (Python Software Foundation) and the pingouin.intraclass_corr function from the Pingouin statistical package (version 0.3.12), based on a two-way mixed-effects model, in terms of consistency for multiple (ICC3,k) and single (ICC3,1) measurements, treating the OCT acquisition modes as fixed raters. Bland-Altman diagrams are provided in S2 Fig for the comparison of inter-raters fixed bias of the average transmitted coherent light, Tcoh(stroma), with ‘Line’ mode used as a reference. The precision of the extracted parameters as a function of number of measurements is illustrated in S3 Fig. (DOCX) [file pone.0291613.s007.docx]

#### **S2. Reliability and precision of the extracted parameters.**

#### The different scan modes provide data of unequal quality (see Supplemental Table S3 for intraclass correlation (ICC) measurement), which results in two trends in reliability for the extracted parameters. On the one hand, our quantification parameters of tissue homogeneity and transparency, namely $B_{r}$, $l_{s},$ and $T_{coh(stroma)}$, have excellent inter-mode reliability in cross-sectional acquisition modes (‘Line’ and ‘Cross’) for the mean of $k$ measurements (p-values << 0.05; respective ICC_3,k_ [$CI_{95}]$ estimates of 0.999 [0.91––1], 0.999 [0.98––1], and 0.999 [0.96––1]) as well as for single measurements (p-values << 0.05; respective ICC_3,1_ [$CI_{95}$] estimates of 0.999 [0.84––1], 0.995 [0.95––1], and 0.999 [0.92––1]). On the other hand, a Tukey Honest Significant Difference (HSD) test revealed a fixed bias for $T_{coh(stroma)}$measurements with pachymetry mapping OCT images (‘Pachy’ and ‘PachyWide’ scan modes; see Supplemental Figure S4 for Bland-Altman comparison of OCT acquisition modes); that bias was significant for ‘Pachy’ mode, which can also be noted in Supplemental Figure S5 where the measurements for the right eye (OD; right panel) are significantly higher in ‘Pachy’ mode than in the other modes.

The precision of the extracted parameters ($\Delta$) using single measurements is derived from the dispersion of the data for reliability testing ($\Delta x=t SD$ with $t=2.26$ being the coefficient given by the Student’s t-distribution for $n=10$ images and a 95% confidence interval), namely $\Delta l_{s}=\pm120 \mu m$ and $\Delta T_{coh(stroma)}=\pm9\%$ for the ‘Line’ scan mode, $\Delta l_{s}=\pm230 \mu m$ and $\Delta T_{coh(stroma)}=\pm18\%$ for the ‘Cross’ mode, $\Delta l_{s}=\pm1500 \mu m$ and $\Delta T_{coh(stroma)}=\pm25\%$ for the ‘Pachy’ mode and $\Delta l_{s}=\pm800 \mu m$ and $\Delta T_{coh(stroma)}=\pm22\%$ for the ‘PachyWide’ mode. This precision could be improved by performing multiple measurements of the same cornea. In ‘Line’ or ‘Cross’ mode, the average coherent transmittance of a given cornea converges within a $\pm5\%$ interval for a sample size of at least 3 similar images (same OCT acquisition mode) and within a $\pm3\%$ interval for a sample size of 5 images or more (see Supplemental Figure S5). The respective results for ‘Pachy’ and ‘PachyWide’ modes are 6 similar images or more for a $\pm5\%$ interval and at least 8 images for a $\pm3\%$ interval. Moreover, we found no significant bias from the removal of the saturation artifact (Pearson’s correlation test between artifact width and $T_{coh(stroma)}$: p-value $\gg0.05$ for every acquisition mode).
