# Supplementary material for: A new method for in vivo assessment of corneal transparency using spectral-domain OCT
Source: PLoS One. 2023 Oct 5;18(10):e0291613. doi: 10.1371/journal.pone.0291613 (PMC10553212; doi:10.1371/journal.pone.0291613)
Supplement: S2 Text — Results for the entire sample (n = 83, all ‘Line’ and ‘Cross’ images) and for the reduced sample (n = 42, images without saturation artifact or with a saturation artifact narrower than 300 μm) are given. (DOCX) [file pone.0291613.s008.docx]

**S7. Shapiro-Wilk normality test results.**

**Shapiro-Wilk normality test of the entire sample (n=83):**

1. Log of $l_{s}$ values, considering up to the 70^th^ percentile. $W=0.96$, p-value = 0.13 > 0.05: log-normality accepted. This distribution is expected since the photon-mean free path can reach several-millimeter values in very transparent tissues (as has been theoretically computed in [^[[1]](#footnote-1)^]);
2. $T_{coh(stroma)}$ values. $W=0.94$, p-value = 0.001 < 0.05: normality rejected. The distribution looks bimodal with major coherent transmittance peaks around 38% and 80% plus a minor peak around 18%.

**Shapiro-Wilk normality test of the reduced sample (n=42):**

1. Log of $l_{s}$ values, considering up to the 70^th^ percentile. $W=0.92$, p-value = 0.02 < 0.05: log-normality rejected. The median has almost doubled with respect to sub-figure (A);
2. $T_{coh(stroma)}$ values. $W=0.93$, p-value = 0.02 < 0.05: normality rejected. The 40% peak has drastically decreased compared to the 80% one, with respect to sub-figure (B).

1. [] Bocheux R. Caractérisation objective et quantitative de la transparence cornéenne par OCT plein champ et microscopie holographique. Ph.D. Thesis, Université Paris-Saclay (COmUE). 2019. Available from: https://pastel.hal.science/tel-02928883 [↑](#footnote-ref-1)
