# Supplementary material for: A new method for in vivo assessment of corneal transparency using spectral-domain OCT
Source: PLoS One. 2023 Oct 5;18(10):e0291613. doi: 10.1371/journal.pone.0291613 (PMC10553212; doi:10.1371/journal.pone.0291613)
Supplement: S1 Table — Intraclass correlation coefficient estimates (ICC3,k and ICC3,1) and their 95% confidence intervals (CI) are shown, with k representing the number of measurements. (DOCX) [file pone.0291613.s009.docx]

| **Parameter** | **Mode**  **(= rater)** | **Measurements** | | | | | | **ICC_3,k_ for the 4 OCT modes** | **p-value** | **95% CI** | **ICC_3,k_ for 'Line' and 'Cross' OCT** | **p-value** | **95% CI** | **ICC_3,1_ for 'Line' and 'Cross' OCT** | **p-value** | **95% CI** |
| --- | --- | --- | --- | --- | --- | --- | --- | --- | --- | --- | --- | --- | --- | --- | --- | --- |
|  |  | **Left eye (OS)** | | | **Right eye (OD)** | | |  |  |  |  |  |  |  |  |  |
|  |  | **mean** | **SD** | **CV** | **mean** | **SD** | **CV** |  |  |  |  |  |  |  |  |  |
| **SNR [dB]** | **Cross** | 18.7 | 0.6 | 3% | 18.7 | 0.6 | 3% | 0.4 | 0.3 | -9.7 – 1 | 0.2 | 0.5 | -529 – 1 | 0.1 | 0.5 | -1 – 1 |
|  | **Line** | 18.1 | 0.5 | 3% | 17.5 | 0.6 | 4% |  |  |  |  |  |  |  |  |  |
|  | **Pachy** | 19.3 | 0.8 | 4% | 19.4 | 0.4 | 2% |  |  |  |  |  |  |  |  |  |
|  | **PachyWide** | 18.8 | 0.6 | 3% | 18.5 | 0.9 | 5% |  |  |  |  |  |  |  |  |  |
| $\boldsymbol{B}_{\boldsymbol{r}}$ | **Cross** | 2.2 | 0.6 | 28% | 3.1 | 0.6 | 18% | 0.8 | 0.1 | -2.3 – 1 | 0.999 | 0.007 | 0.91 – 1 | 0.999 | 0.007 | 0.84 – 1 |
|  | **Line** | 2.1 | 0.5 | 26% | 3.0 | 0.6 | 21% |  |  |  |  |  |  |  |  |  |
|  | **Pachy** | 2.3 | 0.8 | 33% | 2.3 | 0.4 | 18% |  |  |  |  |  |  |  |  |  |
|  | **PachyWide** | 2.5 | 0.6 | 24% | 2.8 | 0.9 | 31% |  |  |  |  |  |  |  |  |  |
| **log(**$\mathcal{l}_{\boldsymbol{s}}$**)** | **Cross** | 6.2 | 0.2 | 3% | 5.9 | 0.3 | 5% | < 0 | 0.7 | -5123 – 0.99 | 0.999 | 0.004 | 0.98 – 1 | 0.995 | 0.004 | 0.95 – 1 |
|  | **Line** | 6.2 | 0.1 | 2% | 5.9 | 0.1 | 2% |  |  |  |  |  |  |  |  |  |
|  | **Pachy** | 6.5 | 0.3 | 5% | 7.2 | 0.5 | 7% |  |  |  |  |  |  |  |  |  |
|  | **PachyWide** | 6.4 | 0.6 | 9% | 5.9 | 0.1 | 1% |  |  |  |  |  |  |  |  |  |
| ***T*_coh(stroma)_** | **Cross** | 41% | 6% | 15% | 28% | 10% | 34% | < 0 | 0.6 | -54 – 1 | 0.999 | 0.005 | 0.96 – 1 | 0.999 | 0.005 | 0.92 – 1 |
|  | **Line** | 42% | 5% | 12% | 29% | 3% | 11% |  |  |  |  |  |  |  |  |  |
|  | **Pachy** | 48% | 10% | 20% | 70% | 12% | 17% |  |  |  |  |  |  |  |  |  |
|  | **PachyWide** | 44% | 17% | 38% | 28% | 3% | 10% |  |  |  |  |  |  |  |  |  |

**S3 Table. Reliability measurements of objective parameters.**

Intraclass correlation coefficient estimates (ICC_3,k_ and ICC_3,1_) and their 95% confidence intervals (CI) are shown, with *k* representing the number of measurements.
